# Using 3dRPC for RNA–protein complex structure prediction

**DOI:** 10.1007/s41048-017-0034-y

**Published:** 2017-02-10

**Authors:** Yangyu Huang, Haotian Li, Yi Xiao

**Affiliations:** Biomolecular Physics and Modeling Group, School of Physics, Huazhong University of Science and Technology, Wuhan, 430074 China

**Keywords:** RNA–protein complex, Tertiary structure, Computational prediction, Docking, Scoring function

## Abstract

3dRPC is a computational method designed for three-dimensional RNA–protein complex structure prediction. Starting from a protein structure and a RNA structure, 3dRPC first generates presumptive complex structures by RPDOCK and then evaluates the structures by RPRANK. RPDOCK is an FFT-based docking algorithm that takes features of RNA–protein interactions into consideration, and RPRANK is a knowledge-based potential using root mean square deviation as a measure. Here we give a detailed description of the usage of 3dRPC. The source code is available at http://biophy.hust.edu.cn/3dRPC.html.

## Introduction

RNA–protein interactions have drawn much attention recently since they might play important roles in many biological processes (Chen and Varani 2005; Glisovic et al. 2008). It was found that most of the human genome could be transcribed into RNAs but only a small fraction of these RNAs was translated into proteins (Cheng et al. 2005), i.e., most RNAs did not undergo translation. These non-coding RNAs perform their biological functions mostly through RNA–protein interactions and forming RNA–protein complexes. As the protein–protein interactions, the three-dimensional structures of RNA–protein complexes are essential to understand the mechanism of RNA–protein interactions. However, experimental determination of three-dimensional structures of RNA–protein complexes is still difficult and time-consuming at present. To solve this problem, computational methods have been proposed to predict the RNA–protein complex structures.

Most algorithms for predicting complex structure consist of two stages: sampling and scoring. The first stage is sampling conformational space and selecting candidates. Since the conformational space is very large, a fast and effective sampling method is required. The second stage is evaluation of the candidates using a ranking or scoring function. Compared to the well-developed methods for protein–protein complex structure prediction (Vakser and Aflalo 1994; Gabb et al. 1997; Chen et al. 2003; Dominguez et al. 2003; Kozakov et al. 2006), those for RNA–protein complexes remain to be developed, which mainly focus on the scoring (Chen et al. 2004; Perez-Cano et al. 2010; Tuszynska and Bujnicki 2011; Li et al. 2012; Huang and Zou 2014), while the sampling methods were borrowed from those for protein–protein complex prediction (Vakser and Aflalo 1994; Gabb et al. 1997; Chen et al. 2003). Recently, we proposed a novel protocol for predicting RNA–protein complex structures—3dRPC (Huang et al. 2013). 3dRPC originally consists of a docking procedure RPDOCK and a scoring function DECK-RP.

RPDOCK is a docking procedure specific to RNA–protein docking. Based on the fact that the atom packing at the RNA–protein interface is different from that at the protein–protein interface (Jones et al. 1999, 2001; Bahadur et al. 2008), RPDOCK applies a new set of parameters to calculate the geometric complementarity. Since the electrostatics plays an important role in RNA–protein interaction(Jones et al. 2001; Kim et al. 2006; Terribilini et al. 2006; Bahadur et al. 2008; Kumar et al. 2008; Perez-Cano et al. 2010; Perez-Cano and Fernandez-Recio 2010), RPDOCK also includes electrostatic effect. RPDOCK also accounts for the stacking interactions between aromatic side chain and bases. The scoring function DECK-RP has been replaced in the updated 3dRPC by RPRANK, a new knowledge-based potential using Root mean square deviation (RMSD) as a measure. The statistical objects of RPRANK are the conformation differences between residue-base pairs. The residue-base pairs are clustered based on the RMSD between each other. Then the energies of the residue-base pair clusters are decided by statistical method based on the number of pairs in each cluster. Different from other statistical potential, this potential does not use distance to classify the residue-base pairs directly. The RMSD-based potential RPRANK has been tested on Zou’s benchmarks (Huang and Zou 2013). The success rate reaches 29.1% for top one and 41.7% for top ten. 3dRPC has been tested on two test sets(Perez-Cano et al. 2012; Huang and Zou 2013) and achieved success rates of 12.1% and 31.9% for top one prediction and 28.8% and 41.7% for top ten, respectively. In the following, we give a detailed description of the usage of 3dRPC.

### 3dRPC

#### Stage 1: rigid-body docking by RPDOCK

RPDOCK is a FFT-based, rigid-body sampling method. The overall process of RPDOCK resembles protein–protein docking algorithm FTDOCK (Gabb et al. 1997). First, the protein is discretized into three-dimensional grid and the RNA is rotated by Euler angles and then discretized into three-dimensional grid. Next, a full translation scan is performed. During the translation scan, top three poses are retained according to the RPDOCK score. Fast Fourier transform is used to accelerate the calculation. The process is repeated until full rotation scan is completed. RPDOCK score is composed of two items: geometric complementarity (GC) and electrostatics (ELEC). The electrostatics is calculated by Coulomb’s formula with a distance-dependent dielectric and the charge is extracted from AMBER force field (Case et al. 2005).

#### Stage 2: scoring by RPRANK

Each presumptive pose generated by RPDOCK is scored by RPRANK in this stage. RPRANK extracts the residue-base pairs within 10 Å, and then the pairs from decoy complexes are compared with standard pairs that are from native structures. If the RMSD between standard pair and decoy pair is less than 6 Å, the energy of decoy pair will be recorded as same as the standard pair. Finally, the energy of the decoy complex is the sum of the energy of pairs.

## Procedure

### 3dRPC installation

1. To download 3dRPC package, visit the 3dRPC webpage (http://biophy.hust.edu.cn/3dRPC.html).

2. Set running environment for 3dRPC. Add the following lines to your “~/.bashrc”:
  “export HOME_3dRPC=/home/XXX/3dRPC/”,
  “export X3DNA=${HOME_3dRPC}/ext/X3DNA/”,
  “export PATH=$PATH:${HOME_3dRPC}/ext/fasta/”.
  Type the command in your terminal:
  “source ~/.bashrc”.

3. Download and install libraries. Three external libraries are required by 3dRPC: FFTW (http://www.fftw.org/download.html), BLAS (http://www.netlib.org/blas/), and LAPACK (http://www.netlib.org/lapack/). The default path of libraries is “${HOME_3dRPC}/lib/”.
**[? TROUBLESHOOTING]**

4. Install FASTA. FASTA is used for sequence alignment in 3dRPC. The source code of FASTA is located on “${HOME_3dRPC}/ext/fasta/”. Users can execute the following command lines to install FASTA:
  “cd ${HOME_3dRPC}/ext/fasta/”,
  “make”.
  After successful installation, an executable file “fasta35” can be found in “${HOME_3dRPC}/ext/fasta/”.

5. Install 3dRPC program from the source code. Run the following command lines given below:
  “cd ${HOME_3dRPC}/source”,
  “make”.
**[? TROUBLESHOOTING]**

### Docking by RPDOCK

**Fig. 1:**
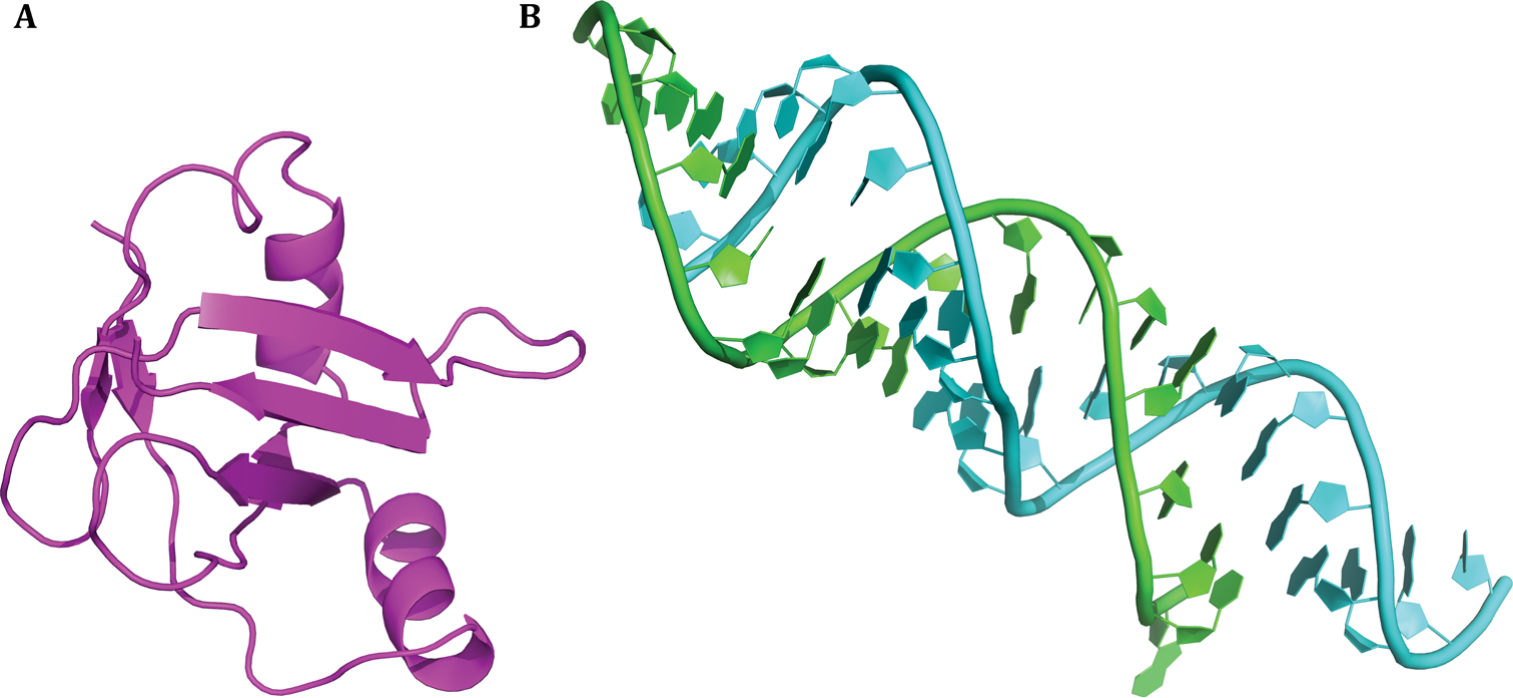
An example of docking. The case is obtained from RNA–protein docking benchmark. The PDB code is 1DFU. Unbound protein (A) and unbound RNA (B) are shown in cartoon presentation

6. Prepare two PDB structures for docking, with one being protein and the other one being RNA. An example is shown in Fig. 1.

7. Prepare the parameter files for RPDOCK. The parameter files must follow the following formats:
  RPDock.receptor = 1DFU_r_u.pdb,
  RPDock.receptor.chain = V,
  RPDock.ligand = 1DFU_l_u.pdb,
  RPDock.ligand.chain = CB,
  RPDock.outfile = 1DFU.out,
  RPDock.grid_step = 1,
  RPDock.out_pdb = 10.
    The parameter files are further explained in Table 1.

**Table 1:** Explanation of parameter files for RPDOCK—“RPDock.par”

RPDock.receptor	File name of protein structure
RPDock.receptor.chain	Chain ID of protein
RPDock.ligand	File name of RNA structure
RPDock.ligand.chain	Chain ID of RNA
RPDock.outfile	Output file name of RPDOCK
RPDock.grid_step	Grid step of RPDOCK, 1 is recommended
RPDock.out_pdb	Number of complexes generated

8. Run RPDOCK by the following command line:
  “$HOME_3dRPC/source/3dRPC -mode 9 -system 9 -par RPDock.par”.
  “RPDock.par” is the parameter file described previously. After docking is finished, RPDOCK will generate an output file “1DFU.out” and a number of docked complexes (“complex1.pdb”, …, “complex*.pdb”). An example of the output files is shown below:

**Table:** 

G_DATA	13	0	−946.00	13	25	1	3	48.0	0.0	0.0
G_DATA	10	0	−897.00	10	25	5	2	36.0	0.0	0.0
G_DATA	14	0	−858.00	14	25	2	3	48.0	0.0	0.0
    Each line represents a docked complex with related information (Table 2). RPDOCK is a rigid-body docking procedure and the docked complexes depend on the translation vector and the rotation angles (Fig. 2).

**Table 2:** Explanation of information contained in the output files of RPDOCK

Column 4	RPDOCK score
Column 6–8	Translation vector
Column 9–11	Rotation angles

**Fig. 2:**
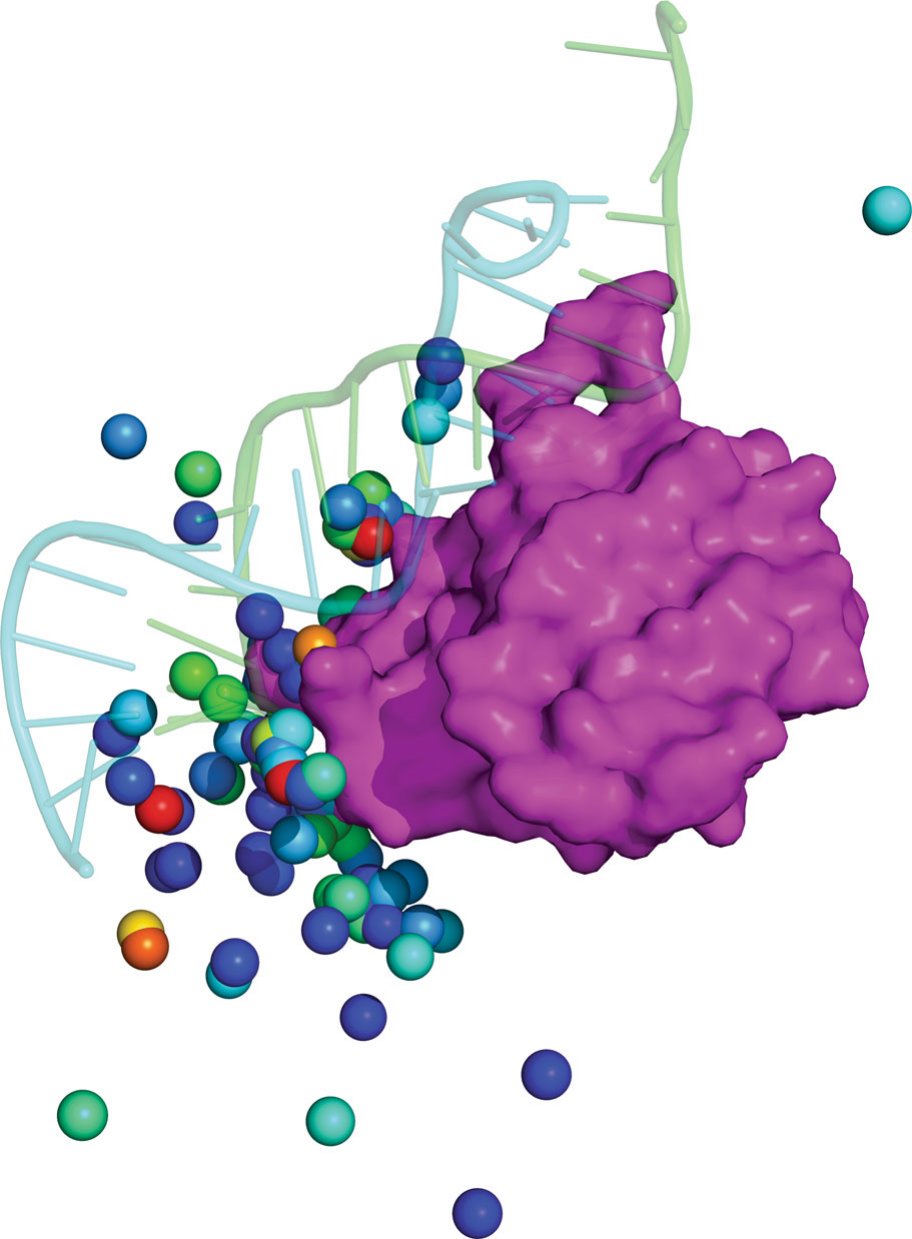
An example of docking. The native complex (1DFU) is shown in cartoon. The centroids of top 100 poses according to RPDOCK score are shown in sphere with rainbow color representing RPDOCK score. The *red color* represents high RPDOCK score

9. Generate complexes by the following command line:
  “$HOME_3dRPC/source/3dRPC -mode 9 -system 8 -par RPDock.par”.
  “RPDock.par” is the same parameter file that is used for docking. Users can change the number of complexes generated.

### Scoring with RPRANK

10. Prepare a list of complex structures to be scored by the following format:

**Table:** 

complex1.pdb	V	CB
complex2.pdb	V	CB
  The first column is the file name of the complex structures, the second column is the chain ID of protein and the last column is the chain ID of RNA.

11. Prepare the parameter file “scoring.par” for scoring:
  list = list,
  out = RMSD.score.

12. Run the command to score the complexes in the list:
  “${HOME_3dRPC}/source/3dRPC -mode 8 -system 9 -par scoring.par”.
    According to the parameter, the output of scoring is saved in the file “RMSD.score”. An example of the output is shown below:

**Table:** 

complex1.pdb	−93.2882
complex2.pdb	−145.628
    The first column is the name of the complex and the second column is the corresponding energy given by RMSD-based score.

### Result analysis of RPDOCK decoy

13. Prepare the parameter file for analysis:
  RPDock.resfile = 1DFU.out,
  RPDock.max_matches = 10,
  native.receptor_pdb_filename = 1DFU_r_b.pdb,
  native.ligand_pdb_filename = 1DFU_l_b.pdb,
  native.receptor.chainid = P,
  native.ligand.chainid = MN,
  decoy.receptor_pdb_filename = 1DFU_r_u.pdb,
  decoy.ligand_pdb_filename = 1DFU_l_u.pdb,
  decoy.receptor.chainid = V,
  decoy.ligand.chainid = CB,
  rmsd.output = 1DFU.rmsd.dat (Table 3).

**Table 3:** Explanation of the parameter files

RPDock.resfile	Output of RPDOCK
RPDock.max_matches	Number of complexes
native.receptor_pdb_filename	Native protein structure
native.ligand_pdb_filename	Native RNA structure
native.receptor.chainid	Chain ID of native protein
native.ligand.chainid	Chain ID of native RNA
decoy.receptor_pdb_filename	Protein structure used for docking
decoy.ligand_pdb_filename	RNA structure used for docking
decoy.receptor.chainid	Chain ID
decoy.ligand.chainid	Chain ID
rmsd.output	Output file of result analysis

14. Run the following command:
  “${HOME_3dRPC}/source/3dRPC -mode 2 -system 0 -par rmsd.par”.
    The “rmsd.par” is the parameter file described in step 15. After the calculation is finished, an outfile, named as “1DFU.rmsd.dat” according to the parameter, will be generated. The output files are formatted as following:

**Table:** 

#Decoy	R_rmsd	L_rmsd	I_rms	fnat	fnon
1	0.744382	34.1629	14.6322	0	1
2	0.744382	32.8772	14.5631	0.0178571	0.964286
  Further explanation of the files is shown in Table 4.

**Table 4:** Explanation of output files

#Decoy	Decoy number
R_rmsd	RMSD of receptor (protein)
L_rmsd	RMSD of ligand (RNA)
I_rms	Interface RMSD
fnat	Native contact fraction
fnon	Non-native contact fraction

**[? TROUBLESHOOTING]**

Step 3: How to install BLAS and LAPACK in Mac?

Open the file “BLAS/make.inc” or “LAPACK/make.inc”, find the line that says: “PLAT = _LINUX” and change it to “PLAT = _MACOS”. Type “make” in your terminal to install BLAS and LAPACK.

Step 5: What can I do if I get error while installing 3dRPC?

Make sure that BLAS, LAPACK and FFTW libraries are successfully installed in your system. Open the file “${HOME_3dRPC}/source/Makefile”, find the line starting with “LAPACK_LIBS” and “BLAS_LIBS”, make sure that the paths of the libraries are correctly assigned.
